# Supplementary material for: Metataxonomics and Metabolomics Profiles in Metabolic Dysfunction-Associated Fatty Liver Disease Patients on a “Navelina” Orange-Enriched Diet
Source: Nutrients. 2024 Oct 18;16(20):3543. doi: 10.3390/nu16203543 (PMC11510614; doi:10.3390/nu16203543)
Supplement: Supplementary file 1 [file nutrients-16-03543-s001.zip › supplementary_figure_S1.pdf]

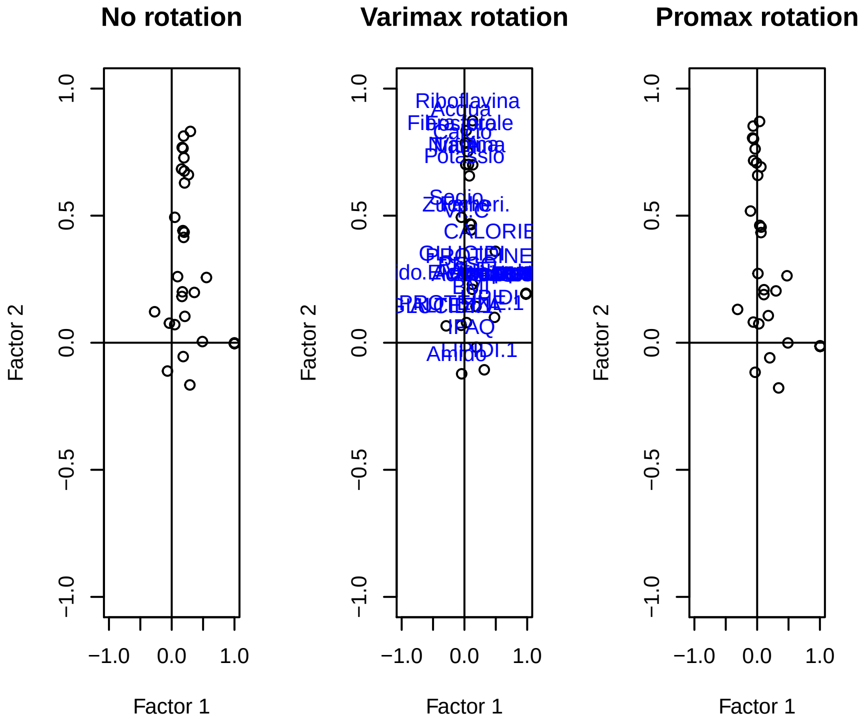

No rotation
Varimax rotation
Promax rotation
Factor 1
Factor 2
1.0
0.5
0.0
−0.5
−1.0
Riboflavina
Acqua
Calcio
Potassio
CALORIE
BMI
IPAQ
LIPIDI.1
Amido

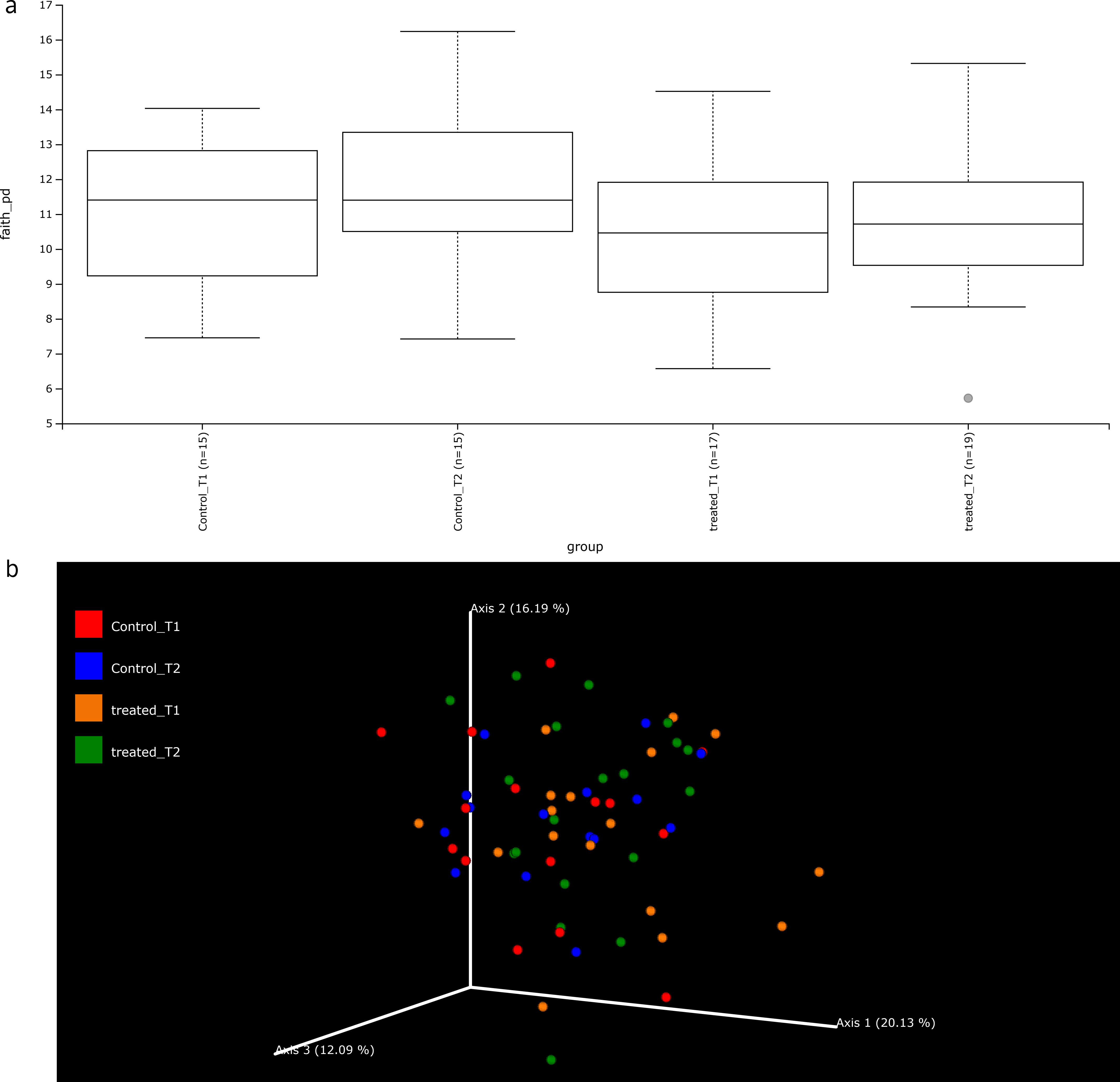
a
faith_pd
17
16
15
14
13
12
11
10
9
8
7
6
5
Control_T1 (n=15)
Control_T2 (n=15)
treated_T1 (n=17)
treated_T2 (n=19)
group
b
Control_T1
Control_T2
treated_T1
treated_T2
Axis 2 (16.19 %)
Axis 1 (20.13 %)
Axis 3 (12.09 %)

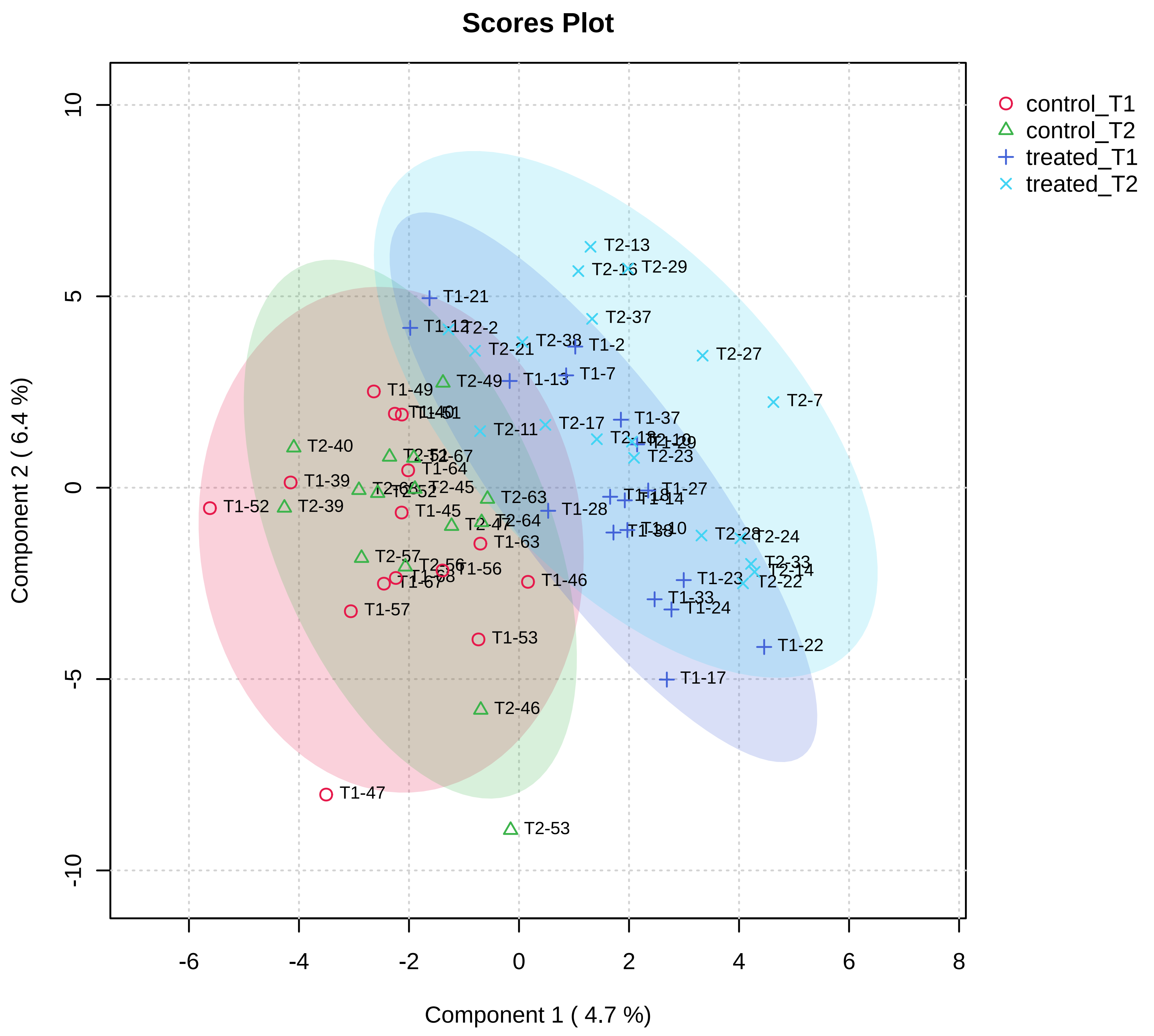

Scores Plot
Component 1 (4.7 %)
Component 2 (6.4 %)
-6
-4
-2
0
2
4
6
8
10
5
0
-5
-10
control_T1
control_T2
treated_T1
treated_T2
T2-13
T2-29
T1-21
T2-37
T2-2
T2-38
T1-2
T2-21
T2-27
T1-13
T1-7
T2-49
T1-49
T2-7
T1-37
T2-11
T2-17
T2-40
T2-23
T1-64
T1-39
T1-27
T2-45
T2-63
T1-52
T2-39
T1-14
T1-28
T1-45
T2-64
T2-47
T1-63
T2-24
T2-57
T1-56
T2-22
T1-46
T1-23
T1-57
T1-33
T1-24
T1-53
T1-22
T1-17
T2-46
T1-47
T2-53
