# Supplementary material for: Metataxonomics and Metabolomics Profiles in Metabolic Dysfunction-Associated Fatty Liver Disease Patients on a “Navelina” Orange-Enriched Diet
Source: Nutrients. 2024 Oct 18;16(20):3543. doi: 10.3390/nu16203543 (PMC11510614; doi:10.3390/nu16203543)
Supplement: Supplementary file 1 [file nutrients-16-03543-s001.zip › Supplementary_Table_S1.pdf]

Gentle Red Wheat	Mandarin	Cauliflower
Sorghum	Mango	Endive
Almonds	Mapo	Potatoes
Nuts	Orange	Spinach
Apple	Papaya	Basil
Apricots	Peas	Chili
Banana	Peppers	Mint
Bergamot	Plums	Origan
Blackcurrant	Pomelo	Parsley
Calamondino or Bitter Mandarin	Pumpkin	Peppermint
Cedar	Raw Red Tomatoes	Rosemary
Chinese Mandarin or Kumquat	Strawberries	Valerian
Clementines	Tangelo	Chinotto
Combava or Kaffir Lime	Tangerines	Citrus Flavored Tea
Finger Limes	Tangor	Fruit Juices
Grapefruit	Artichokes	Fruit Juices (All Types)
Kiwi	Broccoli	Lemonades
Lemon	Brussels sprouts	Red Wine
Lettuce	Cabbage	White Wine
Lime	Cantaloupe	Citrus Jams and Preserves

■	Grains
■	Nuts
■	Fruits
■	Vegetables
■	Herbs/Spices/Condiments
■	Drinks
■	Others
