# Supplementary material for: Metataxonomics and Metabolomics Profiles in Metabolic Dysfunction-Associated Fatty Liver Disease Patients on a “Navelina” Orange-Enriched Diet
Source: Nutrients. 2024 Oct 18;16(20):3543. doi: 10.3390/nu16203543 (PMC11510614; doi:10.3390/nu16203543)
Supplement: Supplementary file 1 [file nutrients-16-03543-s001.zip › Supplementary_Table_S2.pdf]

Supplementary Materials: Table S2. Anthropometric and clinical variables from enrolled patients (n=62) at baseline (T1) and after 28 days of treatment (T2).

**Parameters** *	**Control** ***(n=31)***			**Treated** ***(n=31)***		
	**T1**	**T2**	**p** ^	**T1**	**T2**	**p** ^
Gender (M) (%)	21 (67.74)	--	--	24 (77.42)	--	--
Age (yrs)	50.06±9.77	--	--	51.77±10.31	--	--
Hepatic steatosis parameter						
CAP (dB/m)	327.71±26.42	324.97±30.61	*ns*	325.81±38.01	305.00±59.60	< 0.05
Anthropometric parameters						
Weigh (kg)	91.95±11.42	91.03±11.47	*ns*	91.98±9.96	91.26±9.53	*ns*
BMI (Kg/m²)	32.31±4.14	31.93±4.35	*ns*	32.07±4.25	31.95±4.28	*ns*
FFM (Kg)	62.45±9.50	62.38±10.08	*ns*	58.40±17.89	61.19±15.53	*ns*
FM (Kg)	30.49±10.34	29.37±10.80	*ns*	29.59±15.94	28.99±13.36	*ns*
TBW (L)	45.87±6.95	45.85±7.33	*ns*	43.02±13.49	44.93±11.60	*ns*
ECW (L)	20.08±2.75	19.85±2.89	*ns*	18.19±7.07	19.54±5.28	*ns*
Biochemical parameters						
AST (U/L)	23.48±7.83	22.90±7.45	*ns*	23.42±9.93	24.29±7.29	< 0.05
ALT (U/L)	30.35±15.74	29.93±15.16	*ns*	36.68±23.74	34.93±18.50	*ns*
γGT (U/L)	29.71±10.78	28.29±10.31	*ns*	40.48±23.01	34.22±21.68	< 0.05
Alkaline Phosphatase (U/L)	69.77±21.00	71.58±21.42	*ns*	67.64±19.99	68.74±19.88	*ns*
Total Cholesterol (mg/dL)	183.26±42.88	186.03±35.94	*ns*	202.29±40.25	193.39±40.83	*ns*
HDL (mg%)	51.44±10.43	51.80±11.94	*ns*	47.10±13.15	47.53±10.74	*ns*
LDL (mg/dL)	119.18±39.86	121.22±39.23	*ns*	132.11±37.64	130.94±35.58	*ns*
Triglycerides (mg/dL)	121.45±70.41	112.29±70.34	*ns*	132.10±53.32	123.06±53.55	*ns*
Glucose (mg/GI)	94.71±9.45	95.71±9.22	*ns*	101.00±21.62	99.59±26.83	*ns*
Insulin (μUI/mL)	15.24±7.03	15.53±8.21	*ns*	16.08±8.55	16.42±9.06	*ns*
HOMA test	3.58±2.13	3.63±2.22	*ns*	4.10±2.50	4.19±2.94	*ns*

* As mean and standard deviation for continuous variables, and as frequency and percentage (%) for categories. ^ Wilcoxon matched-pairs signed-rank test.
Abbreviations: CAP, Controlled Attenuation Parameter; BMI, Body Mass Index; FFM, Free Fat Mass; FM, Fat Mass; TBW, Total Body Water; ECW, Extracellular Water; AST, Aspartate Aminotransferase; ALT, Alanine Transaminase; γGT, Gamma-Glutamyl Transferase; HDL, High-Density Lipoprotein; LDL, Low-Density Lipoprotein; HOMA, Homeostatic Model Assessment.
