# Supplementary figures and images for: Metataxonomics and Metabolomics Profiles in Metabolic Dysfunction-Associated Fatty Liver Disease Patients on a “Navelina” Orange-Enriched Diet
Source: Nutrients. 2024 Oct 18;16(20):3543. doi: 10.3390/nu16203543 (PMC11510614; doi:10.3390/nu16203543)

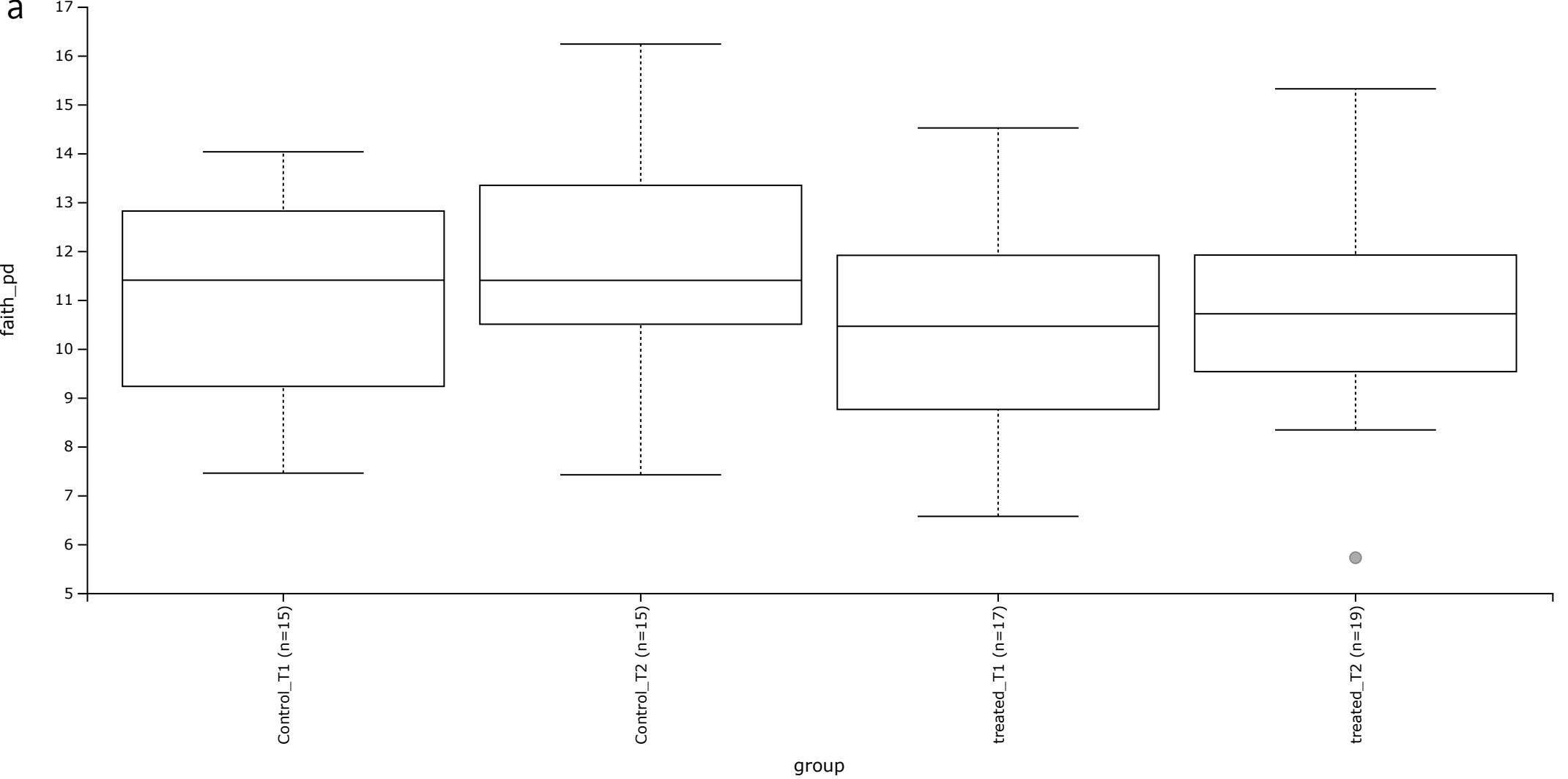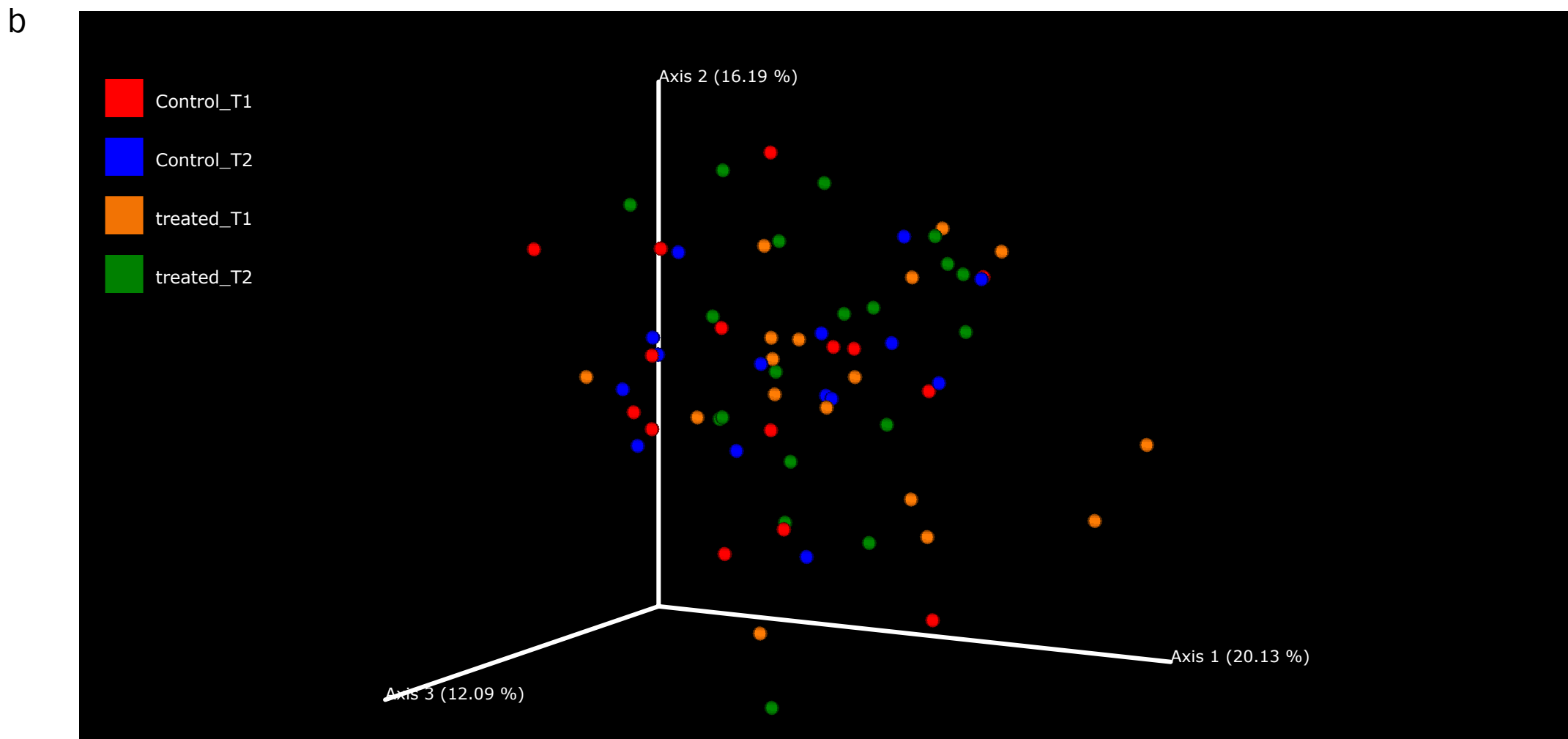

Supplement: Supplementary file 1 [file nutrients-16-03543-s001.zip › supplementary_figure_S2.pdf]

# Scores Plot

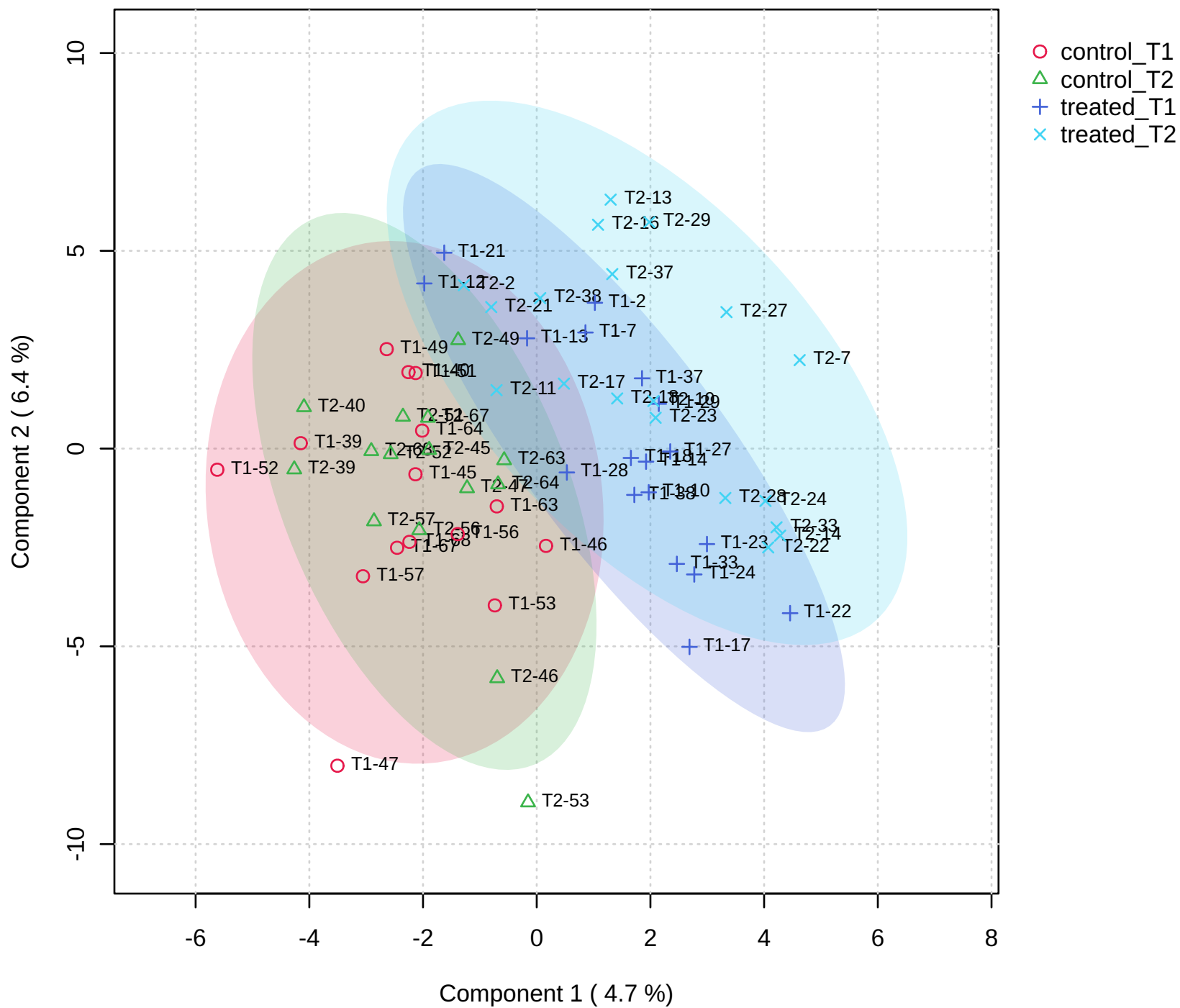

Supplement: Supplementary file 1 [file nutrients-16-03543-s001.zip › Supplementary_figure_S3.pdf]

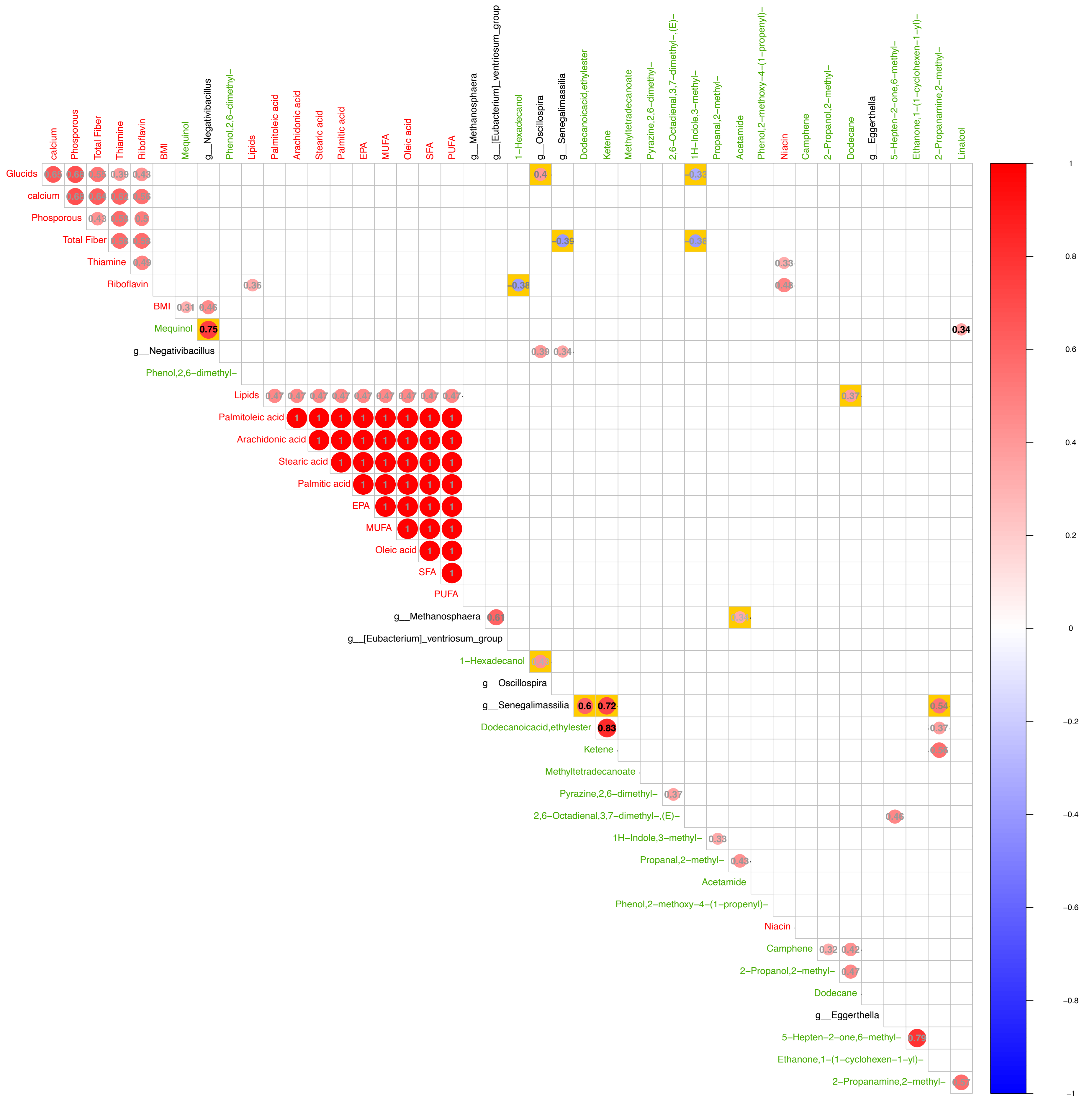

Supplement: Supplementary file 1 [file nutrients-16-03543-s001.zip › Supplementary_figure_S4.pdf]
